# Imaging and fluid biomarkers for prognostic stratification in cerebral amyloid angiopathy

**DOI:** 10.1007/s00415-026-13626-2

**Published:** 2026-01-21

**Authors:** Dandan Wang, Shuxian Lv, Yuqing Wei, Xingquan Zhao

**Affiliations:** 1 Department of Neurology, Beijing Tiantan Hospital, Capital Medical University, No.119, South 4th Ring Road, Fengtai District, Beijing, 100070 China; 2 China National Clinical Research Center for Neurological Diseases, Beijing, China; 3 Department of Neurology, Weill Institute for Neurosciences, Edward and Pearl Fein Memory and Aging CenterUniversity of California, San Francisco, San Francisco, CA USA

**Keywords:** Cerebral amyloid angiopathy, Imaging biomarker, Fluid biomarker, Prognosis

## Abstract

Cerebral amyloid angiopathy (CAA) is a common small vessel disease characterized by Aβ deposition in cortical and leptomeningeal arteries, leading to lobar intracerebral hemorrhage and vascular cognitive impairment. Despite advances in diagnosis, prognosis remains highly heterogeneous, encompassing risks of recurrent hemorrhage and progressive cognitive decline. This review summarizes recent developments in imaging and fluid biomarkers for prognostic stratification in CAA. Imaging markers, including advanced MRI and molecular PET techniques, have evolved from traditional hemorrhagic indicators, such as cerebral micro-bleeds (CMBs) and cortical superficial siderosis (cSS), to non-hemorrhagic including white matter hyper-intensities (WMHs), and enlarged perivascular spaces (ePVS), which sensitively capture microstructural damage after using quantitative measures. Fluid biomarkers provide dynamic insights into vascular and neuronal injury, including altered plasma Aβ42/Aβ40 ratios, MMPs/TIMPs balance, and elevated neuro-filament light chain (NfL) and glial fibrillary acidic protein (GFAP) levels. Integrating these multi-modal indicators may enable individualized prediction of hemorrhagic and cognitive outcomes and inform precision management strategies. Future research should standardize quantification methods and validate multi-modal models across diverse CAA phenotypes to advance toward personalized prognostic frameworks.

## Introduction

Cerebral amyloid angiopathy (CAA) is a prevalent, age-related cerebral small vessel disease which involves gradual Aβ accumulation along cortical and leptomeningeal vessel walls. CAA is a predominant cause of lobar hemorrhage among elderly individuals, associated with significant mortality, morbidity, and high rates of recurrence [1]. Beyond acute hemorrhage, CAA is also a crucial pathological substrate for progressive cognitive impairment and transient focal neurological episodes (TFNE) [2]. Recent advancements in neuroimaging, notably the introduction of the Boston Criteria v2.0, have incorporated non-hemorrhagic markers like white matter hyper-intensities (WMHs) to significantly improve the ante-mortem diagnostic accuracy of CAA [3]. Despite these diagnostic leaps, CAA management faces two major clinical challenges: first, the difficulty of individualizing antithrombotic therapy to balance the risk of intracranial hemorrhage (ICH) against the need to prevent ischemic events in a high-risk population [4, 5]; and second, the presence of considerable prognostic heterogeneity, with phenotypes ranging from a predilection for recurrent hemorrhage to one dominated by progressive dementia. This heterogeneity severely complicates overall prognostic assessment and intervention strategy design [6, 7]. Therefore, this review aims to summarize and integrate the latest research advances in neuroimaging and fluid biomarkers, with the goal of establishing a multi-modal integration model for more precisely stratifying CAA patients' hemorrhagic and cognitive risks, thereby informing individualized clinical management.

## Prognostic challenges: the need for multi-modal stratification

CAA prognostic risk assessment currently relies heavily on traditional hemorrhagic magnetic resonance imaging (MRI) markers, particularly the number and location of cerebral micro-bleeds (CMBs) [8–10], but this approach has significant limitations. CMBs only represent the consequence of vessel rupture and hemorrhage, rather than the precursor of active pathology or potential vascular fragility, making them a lagging indicator that fails to capture the chronic pathophysiological activity of the disease [11]. Moreover, CMBs are mainly associated with bleeding risk but have limited predictive ability for CAA-related cognitive decline (CAA-CD) or stroke-like episodes caused by cortical superficial siderosis (cSS), resulting in insufficient sensitivity when assessing CAA-CD risk [1, 12].

The long-term prognosis and disease progression of patients with CAA are highly heterogeneous, showing significant prognostic heterogeneity. While some patients are primarily at risk for recurrent ICH, others present predominantly with progressive cognitive decline and a mounting burden of non-hemorrhagic imaging markers [13]. Traditional imaging struggles to accurately differentiate these two phenotypes, a distinction crucial for treatment: the former may require stricter antithrombotic management, while the latter necessitates considering potential Aβ clearance therapies.

This heterogeneity extends from patients at risk for recurrent hemorrhage to those with predominant cognitive decline, underscoring the need for a more refined, phenotype-specific stratification framework. With the advent of Aβ-targeted immunotherapies, the importance of CAA prognostic stratification has become paramount. CAA is the primary risk factor for Amyloid-Related Imaging Abnormalities (ARIA) -Hemorrhage (ARIA-H). Although baseline CMBs burden is a key factor in predicting ARIA-H risk, it remains a lagging indicator. Thus, there is an urgent need for a multi-modal prognostic model that can both accurately screen high-risk patients before treatment and dynamically monitor early signals of vascular fragility and axonal injury during therapy, thereby informing the safer evaluation of Aβ-targeted therapies [14].

To provide a structured overview of the diverse imaging and fluid biomarkers discussed below in this review, major biomarker categories, representative measures, and their prognostic relevance in cerebral amyloid angiopathy are summarized in Table 1.

**Table 1:** Key imaging and fluid biomarkers for prognostic stratification in cerebral amyloid angiopathy

Biomarker category	Specific biomarker	Key study (year)	Study design/cohort	Prognostic outcome	Main finding	Clinical relevance
Imaging-Hemorrhagic markers	Lobar CMBs burden; cSS	Greenberg et al., 2004 (Stroke) [15]; Roongpiboonsopit et al., 2016(Neurology) [16]; Charidimou et al., 2013 (Neurology) [17]; Rodrigues et al., 2018 (Lancet Neurol) [18]	Longitudinal cohort studies; diagnostic model development	ICH recurrence; future hemorrhagic risk	Higher lobar CMBs burden and presence of cSS predict recurrent lobar intracerebral hemorrhage, with greater cSS extent conferring higher risk and informing hemorrhagic risk stratification frameworks	Hemorrhagic risk
Imaging-on-hemorrhagic markers	WMHs; centrum semiovale ePVS	van Rooden et al., 2016 (Stroke) [19]; Charidimou et al., 2016 (Neurology) [20]; Makkinejad et al., 2024 (J Am Heart Assoc) [23]; Charidimou et al., 2017 (Neurology) [24]; Perosa et al., 2022 (Acta Neuropathol) [27]	Longitudinal and cross-sectional cohorts; MRI–neuropathology correlation	Cognitive impairment; disease severity	Greater WMHs burden is associated with cognitive impairment in CAA beyond hemorrhagic lesion burden. High centrum semiovale ePVS burden is more frequent in CAA and reflects impaired perivascular clearance	Cognitive outcome
Imaging-Molecular imaging (PET)	Amyloid PET; Tau PET	Gurol et al., 2016 (Neurology) [28]; Romoli et al., 2024 (Neurology) [29]; Schoemaker et al., 2021 (Neurology) [30]	Prospective and multicenter cohort studies	Hemorrhagic risk; cognitive impairment; phenotypic classification	Amyloid PET aids CAA classification and phenotype refinement beyond conventional MRI markers, with cohort-level associations with hemorrhagic risk. Tau PET primarily reflects concomitant neurodegenerative pathology and relates more closely to cognitive impairment than hemorrhagic risk	Both
Imaging-Advanced/emerging markers	Diffusion MRI: PSMD; free water imaging; Functional/dynamic MRI: arterial spin labeling (ASL), cerebrovascular reactivity (CVR), dynamic contrast-enhanced MRI (DCE-MRI/BBB leakage)	Horn et al., 2023 (Front Neurosci) [31]; Farias Da Guarda et al., 2025 (Neurology) [32]; Zhang et al., 2012 (J Alzheimers Dis) [33]; Switzer et al., 2020 (Neurology) [34]; van den Brink et al., 2025 (Neurology) [35]	Cross-sectional and longitudinal cohort studies; exploratory imaging studies	Cognitive impairment; disease severity; microvascular dysfunction	PSMD and free water imaging capture diffuse white matter microstructural injury and associate with cognitive impairment and disease severity in CAA. Functional and dynamic MRI techniques assess perfusion, vascular reactivity, and BBB integrity, providing complementary information beyond static lesion burden	Cognitive outcome
Fluid biomarkers	Amyloid-related plasma biomarkers (Aβ42/Aβ40, Aβ40); Vascular remodeling/inflammatory markers (MMPs/TIMPs); Neuronal and glial injury markers (NfL, GFAP); Exploratory biomarkers (extracellular vesicles, proteomic/metabolomic signatures)	Muir et al., 2025 (Alzheimers Dement) [36]; Bornebroek et al., 2003 (Neurobiol Dis) [37]; Vervuurt et al., 2023 (Alzheimers Res Ther) [39]; Cheng et al., 2020 (Aging) [40]; Rasing et al., 2024 (Alzheimers Res Ther) [41]	Cross-sectional and longitudinal cohorts; plasma and CSF biomarker studies	Hemorrhagic risk; cognitive decline; disease progression	Amyloid-related plasma biomarkers reflect vascular amyloid burden and clearance dynamics, with inconsistent prediction of hemorrhagic events. Vascular remodeling markers (MMPs/TIMPs) indicate vessel wall instability. Neuronal and glial injury markers (NfL, GFAP) associate with cognitive decline and disease progression, while exploratory biomarkers remain investigational	Both

*CMBs* cerebral micro-bleeds, *ICH* intracerebral hemorrhage, *cSS* cortical superficial siderosis, *MRI* magnetic resonance imaging, *WMHs* white matter hyper-intensities, *CAA* cerebral amyloid angiopathy, *ePVS* enlarged perivascular spaces, *PET* positron emission tomography, *ASL* arterial spin labeling, *CVR* cerebrovascular reactivity, *DCE-MRI* dynamic contrast-enhanced magnetic resonance imaging, *BBB* blood–brain barrier, *PSMD* peak width of skeletonized mean diffusivity, *Aβ* amyloid-β, *MMPs* matrix metalloproteinases, *TIMPs* tissue inhibitors of metalloproteinases, *NfL* neuro-filament light chain, *GFAP* glial fibrillary acidic protein

## Imaging cornerstone: advanced quantification of chronic CAA injury

Hemorrhagic imaging markers remain the cornerstone of risk stratification in CAA. Lobar CMBs and cSS are well-established MRI features reflecting vascular amyloid fragility and are strongly associated with the risk of ICH and hemorrhagic recurrence [15, 16]. Importantly, the extent of cSS conveys additional prognostic information, with multifocal or disseminated cSS conferring a substantially higher risk of future lobar intracerebral hemorrhage compared with focal involvement [17]. Together, these hemorrhagic markers not only reflect underlying vascular amyloid pathology but also provide incremental predictive information regarding ICH risk in CAA. In addition, hemorrhagic risk stratification frameworks such as the Edinburgh criteria integrate the extent of cSS and lobar CMBs burden to estimate future intracerebral hemorrhage risk in patients with CAA [18].

Beyond hemorrhagic features, imaging markers increasingly inform CAA diagnosis and prognostic stratification, with the focus shifting from purely hemorrhagic markers to the precise quantification of chronic vascular pathological injury [3]. The increased burden of WMHs is a prognostic indicator in CAA independent of CMBs, and is highly associated with cognitive impairment [19], with longitudinal cohort data showing that greater WMH burden reflects chronic small vessel injury in CAA and remains informative beyond hemorrhagic lesion burden [20]. Pathologically, WMHs reflect CAA-induced blood–brain barrier (BBB) damage, chronic hypoperfusion, and secondary demyelination [21, 22], supported by neuropathological studies demonstrating that WMH burden is significantly associated with cortical vascular Aβ severity, rather than arteriolosclerosis, in definite CAA [23]. Beyond parenchymal white matter injury, growing evidence suggests that CAA also disrupts perivascular clearance pathways, providing a complementary non-hemorrhagic dimension for prognostic assessment. The quantification of enlarged perivascular spaces (ePVS) is also increasingly important in CAA prognosis. In a large cohort of spontaneous ICH, centrum semiovale ePVS were more frequent in CAA than in hypertensive arteriopathy and independently associated with lobar CMBs and cSS, supporting their relative specificity for CAA-related small vessel pathology [24]. A high burden of ePVS is considered indirect imaging evidence of Aβ clearance pathway impairment and glymphatic system dysfunction in CAA [25, 26]. MRI-guided neuropathological studies have shown that ePVS reflects dilated perivascular compartments around cortical perforating arterioles and correlates with vascular Aβ severity rather than arteriolosclerosis or nonspecific aging-related changes [27]. Together, these findings indicate that ePVS capture clearance-related microvascular dysfunction complements WMHs-based measures of parenchymal injury, reinforcing their role as a structural imaging biomarker relevant to CAA progression and prognosis.

Molecular imaging with positron emission tomography (PET) enables in vivo visualization of cerebral amyloid burden and has become an increasingly available and valuable tool in the assessment of cerebral amyloid angiopathy, with characteristic cortical tracer retention reflecting vascular amyloid-β deposition and cohort data suggesting its utility in refining CAA-predominant small vessel disease phenotypes including associations with hemorrhagic risk, and risk stratification beyond conventional MRI markers [28, 29]. Tau PET in CAA, by contrast, has primarily been applied to characterize co-existing neurodegenerative pathology, with emerging evidence indicating that regional tau burden is more closely related to cognitive impairment than to hemorrhagic risk, supporting its complementary and exploratory role in CAA phenotyping [30]. Together, PET imaging provides a molecular context for MRI-defined hemorrhagic and non-hemorrhagic vascular injury, highlighting the value of integrated multi-modal imaging approaches for comprehensive diagnostic and prognostic assessment in CAA.

Beyond conventional structural MRI and PET markers, a growing number of advanced MRI-based quantitative biomarkers have been proposed to capture microstructural and functional aspects of small vessel disease in CAA. Diffusion-derived metrics, including peak width of skeletonized mean diffusivity (PSMD) and free water imaging, provide sensitive measures of diffuse white matter microstructural injury and extracellular fluid changes, and have been associated with cognitive impairment and disease severity in CAA [31, 32]. In parallel, functional and dynamic imaging techniques, such as arterial spin labeling, dynamic contrast-enhanced MRI, and cerebrovascular reactivity measures, enable in vivo assessment of cerebral perfusion, blood–brain barrier integrity, and vascular reactivity, and have been increasingly applied to probe microvascular dysfunction in amyloid-related and CAA-spectrum small vessel disease, capturing aspects not reflected by static lesion burden alone [33–35]. Although these approaches remain primarily confined to research settings, they hold promise for refining phenotypic characterization and prognostic stratification in CAA when integrated with established hemorrhagic, non-hemorrhagic, and molecular imaging markers.

## Fluid biomarkers: reflecting dynamic pathology and neuronal injury

Fluid biomarkers provide complementary and time-sensitive insights into the vascular pathology, vessel wall instability, and downstream tissue injury in CAA, capturing dynamic disease processes that evolve across different stages and are not fully reflected by static imaging markers. In CAA, amyloid-related plasma biomarkers primarily reflect vascular amyloid burden and clearance dynamics. The plasma Aβ42/Aβ40 ratio is typically lower than in healthy controls, reflecting impaired Aβ clearance and deposition in the vessel wall [36]. The prognostic value of plasma Aβ40 for CAA hemorrhagic events and recurrence remains inconclusive. Some studies report significantly lower Aβ40 levels in presymptomatic Dutch type CAA carriers, leading to the hypothesis that Aβ40 levels may initially decrease and subsequently rise in later hemorrhagic stages of CAA as the vascular burden evolves [37, 38].

Beyond amyloid-related measures, circulating markers of vascular remodeling and inflammation may capture vessel wall instability and ongoing structural degradation in CAA. Changes in the ratio of matrix metalloproteinases (MMPs) and tissue inhibitors of metalloproteinases (TIMPs) reflect the activity of vessel wall remodeling and extracellular matrix degradation in CAA, serving as potential dynamic indicators for predicting vascular fragility and hemorrhage risk, particularly at the level of cerebrospinal fluid measurements [38, 39].

In contrast, biomarkers of neuronal and glial injury primarily reflect downstream tissue damage resulting from chronic vascular pathology in CAA. Plasma neuro-filament light Chain (NfL) is a sensitive general marker of axonal injury. In CAA patients, elevated plasma NfL is significantly associated with an accelerated rate of cognitive decline, making it an independent biomarker for predicting CAA-CD progression [36, 40]. Glial fibrillary acidic protein (GFAP), a marker of astrocyte activation and damage, is closely linked to CAA pathology, BBB leakage, and the CAA-related inflammation (CAA-RI) phenotype. GFAP is valuable in CAA because it may reflect active inflammatory reactions in the vessel wall, and may aid in the identification of inflammatory activity in CAA, although its clinical utility for early diagnosis or treatment guidance requires validation from robust longitudinal studies [10, 38, 41].

Beyond these established amyloid- and injury-related fluid biomarkers, emerging exploratory approaches are being investigated in CAA. Among these, extracellular vesicles (EVs), including exosomes, have attracted interest as a potential source of novel biomarkers, as they can carry amyloid-related and inflammatory molecular signatures derived from the neurovascular unit [42, 43]. In parallel, circulating markers of vascular inflammation and BBB dysfunction, as well as unbiased proteomic or metabolomic profiling approaches, are being explored to identify molecular signatures associated with CAA severity and progression [35, 38, 44]. At present, these biomarkers remain largely exploratory and require systematic validation in longitudinal cohorts.

## Multi-modal integration: a refined prognostic framework

Taken together, hemorrhagic risk in CAA is best captured by structural imaging markers, such as cSS and lobar CMBs, whereas cognitive trajectories are more closely linked to non-hemorrhagic imaging injury and neuronal damage biomarkers. Emerging quantitative MRI and fluid markers provide complementary insights into disease activity beyond conventional imaging. Integrating these modalities offers a framework for refined prognostic stratification although further validation in longitudinal and multicenter cohorts is required. These observations provide the rationale for a multi-modal integration framework that jointly considers vascular rupture risk and neurodegenerative progression in CAA.

**Fig. 1:**
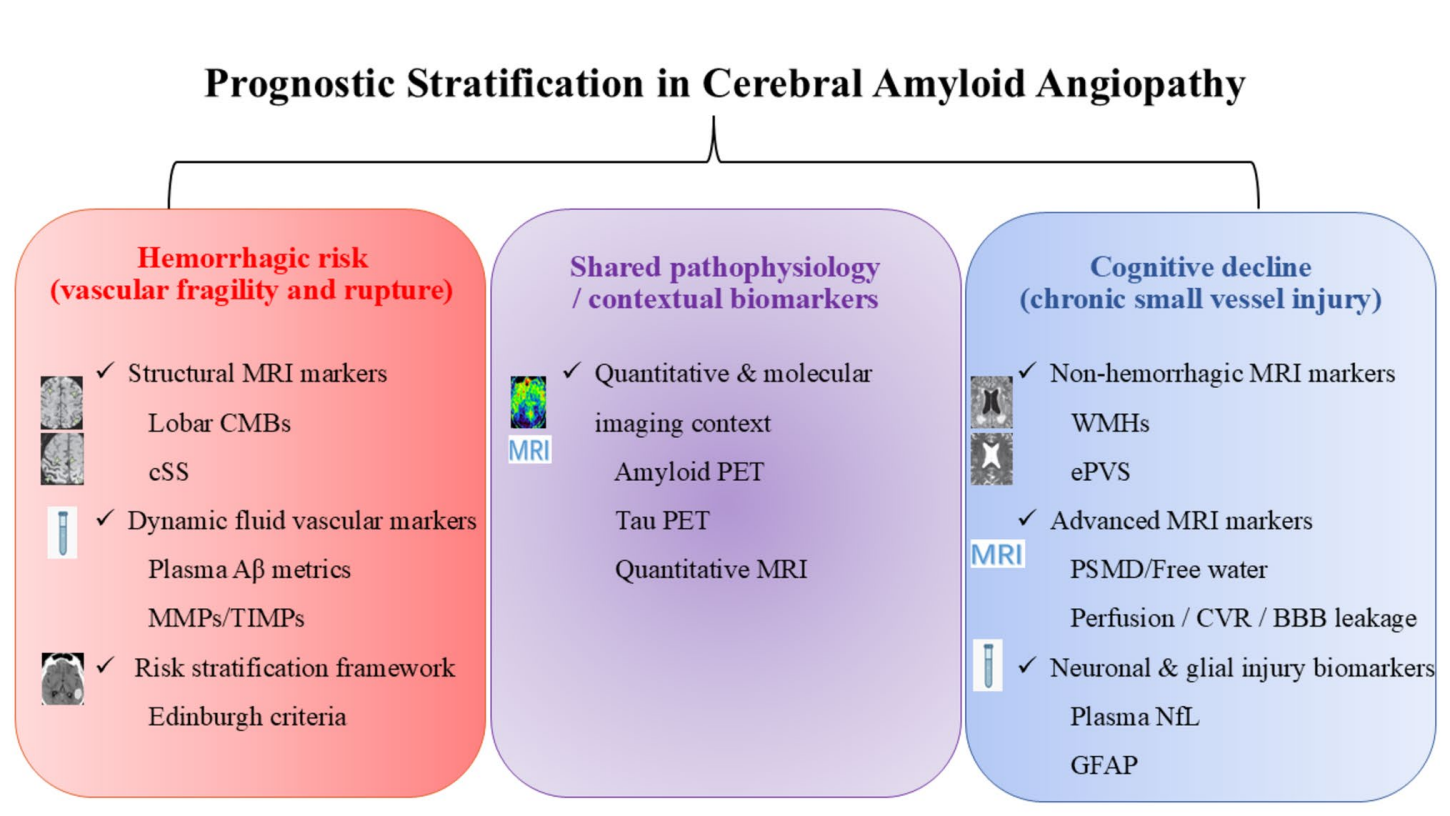
Integration of multi-modal biomarkers for prognostic stratification in cerebral amyloid angiopathy. Some of the selected neuroimaging illustrations are adapted from published work by Marco Duering and colleagues (Duering M, et al. Neuroimaging standards for research into small vessel disease-advances since 2013. Lancet Neurol. 2023 Jul;22(7):602–618). Biomarkers are organized according to their predominant association with hemorrhagic or cognitive outcomes, while recognizing substantial pathophysiological overlap

The core goal of the multi-modal integration model is to simultaneously evaluate vascular re-rupture risk and neurofunctional decline risk. Figure 1 schematically illustrates the integration of imaging and fluid biomarkers across these two partially overlapping prognostic dimensions for predicting hemorrhagic and cognitive outcomes in cerebral amyloid angiopathy.

For prognostic stratification, predicting vascular events requires structural markers (CMBs burden, cSS) as the foundation, combined with dynamic vascular fragility markers, such as altered plasma Aβ metrics and MMPs/TIMPs ratio, which may provide complementary information on vessel wall remodeling and bleeding susceptibility [39, 45]. Simultaneously, predicting cognitive decline risk requires chronic imaging injury markers (advanced WMHs quantification, like PSMD; ePVS burden, ideally with functional imaging validation) as indicators of chronic pathological load, combined with markers of neuronal injury and glial activation, namely sustained elevation of plasma NfL and GFAP, which have been associated with axonal injury and cognitive trajectories [46, 47].

The potential role of this multi-modal framework in the context of Aβ immunotherapy remains exploratory. CAA is the primary risk factor for ARIA-related hemorrhage, making precise risk stratification a prerequisite for ensuring treatment safety [48]. Prior to initiating treatment, a risk score combining structural markers (CMBs burden) and dynamic vascular markers (e.g., Aβ40, GFAP) could be constructed to better characterize hemorrhagic vulnerability. In addition to these imaging and fluid biomarkers, genetic factors are crucial modifiers for predicting ARIA risk. The APOE epsilon 4 genotype should be included as a core weighting factor to adjust the baseline probability of ARIA occurrence post-treatment [49]. Whether dynamic changes in plasma biomarkers, such as NfL and GFAP, can provide complementary information to MRI during immunotherapy requires further investigation. At present, robust evidence supporting biomarker-guided treatment modification is lacking. The ultimate goal is to establish a weighted scoring system to enable truly individualized treatment decisions, which should be regarded as a long-term research objective rather than a current clinical standard [50].

## Outlook and challenges: toward the era of precision medicine for CAA

As CAA encompasses a continuum from asymptomatic microangiopathy to recurrent lobar hemorrhage and progressive dementia, translating multi-modal biomarkers into clinical tools requires validation across the full disease spectrum. Despite significant advances in neuroimaging and fluid biomarkers, several critical challenges remain in translating these findings into routine clinical practice. On the imaging front, newer MRI markers (e.g., advanced quantification of ePVS and functional imaging) are not yet standardized, lacking uniform quantitative methods and validated clinical cut-off values, which limits their application in multicenter studies and the comparability of results [51–53]. Regarding fluid biomarkers, while NfL and GFAP show strong prognostic capability, they lack disease-specific specificity. Their elevation may reflect various neurodegenerative or vascular injuries. Clinical adoption requires establishing age-specific and comorbidity-specific normal reference ranges to accurately isolate the unique pathological contribution from CAA [54–56].

Future research should concentrate on three interconnected areas: First, high-resolution and functional imaging (for example high-field MRI and DCE-MRI) may help to more precisely characterize BBB permeability changes and glymphatic/clearance pathway dysfunction, which are emerging as potential functional risk biomarkers [57, 58]. The second area should aim at identifying molecular injury signals that are more specific to CAA (for example unique Aβ isoforms/cleavage products or molecular changes linked to APOE-targeted therapeutics [59]. The final goal is to link multi-modal risk stratification results with personalized treatment. For individuals characterized by a high hemorrhagic risk profile, management strategies may prioritize strict blood pressure control, while the use of antithrombotic therapy requires careful risk stratification rather than uniform avoidance. Importantly, hemorrhagic risk associated with antithrombotic therapy in CAA is heterogeneous: antiplatelet therapies are not invariably associated with excessive intracerebral hemorrhage risk in all patients, whereas anticoagulation confers a substantially higher and more variable hemorrhagic risk. Identifying individuals at particularly high risk from anticoagulation therefore represents a critical clinical challenge. In selected high-risk populations, alternative strategies such as left atrial appendage occlusion have emerged as potential options to reduce ischemic stroke risk while limiting exposure to long-term anticoagulation [60]. Importantly, hemorrhagic and cognitive trajectories in CAA are not mutually exclusive, and many patients exhibit overlapping vascular and neurodegenerative features across the disease course. In this context, individuals with prominent cognitive vulnerability may represent a population of interest for investigational studies targeting amyloid clearance or related pathways, provided that vascular amyloid burden and hemorrhagic risk are carefully considered. Current evidence regarding anti-amyloid therapies in the presence of CAA-related imaging changes remains limited, and the risk of amyloid-related imaging abnormalities underscores the need for cautious evaluation within controlled trial settings. In addition, for the inflammatory subtype of CAA (CAA-RI), evolving evidence supports a distinct biological and clinical profile. Future work should explore integrating imaging and fluid inflammatory markers (such as GFAP or immune-related immune signatures) to improve phenotypic characterization and risk assessment, rather than to directly guide immunosuppressive treatment decisions. In conclusion, prognostic stratification in CAA is shifting from isolated hemorrhagic markers toward integrated, biology-informed multi-modal frameworks, although clinical translation will require rigorous longitudinal validation.

## Data Availability

All data analyzed during this study are included in this article. Further inquiries can be directed to the corresponding authors.
